# Reticulon 2 promotes gastric cancer metastasis via activating endoplasmic reticulum Ca^2+^ efflux-mediated ERK signalling

**DOI:** 10.1038/s41419-022-04757-1

**Published:** 2022-04-15

**Authors:** Shushu Song, Bo Liu, Xiaoqing Zeng, Yingying Wu, Hao Chen, Hao Wu, Jianxin Gu, Xiaodong Gao, Yuanyuan Ruan, Hongshan Wang

**Affiliations:** 1 Department of Biochemistry and Molecular Biology, School of Basic Medical Sciences, Fudan University, Shanghai, P. R. China; 2 Department of Liver Surgery, Liver Cancer Institute, Zhongshan Hospital, Fudan University, Shanghai, P. R. China; 3 NHC Key Laboratory of Glycoconjugates Research, Fudan University, Shanghai, P. R. China; 4 Department of Gastroenterology, Zhongshan Hospital, Fudan University, Shanghai, P. R. China; 5 Department of General Surgery, Zhongshan Hospital, Fudan University, Shanghai, P. R. China; 6 Department of General Surgery, Zhongshan Hospital Wusong Branch, Fudan University, Shanghai, P. R. China

**Keywords:** Gastric cancer, Calcium signalling

## Abstract

Gastric cancer ranks fourth for mortality globally among various malignant tumours, and invasion and metastasis are the major reason leading to its poor prognosis. Recently, accumulating studies revealed the role of reticulon proteins in cell growth and transmigration. However, the expression and biological function of reticulon proteins in human gastric cancer remain largely unclear. Herein, we explored the potential role of reticulon 2 (RTN2) in the progression of gastric cancer. Tissue microarray was used to determine the expression levels of RTN2 in 267 gastric cancer patients by immunohistochemistry. Gastric cancer cell lines were utilised to examine the influences of RTN2 on cellular migration and invasion abilities, epithelial-to-mesenchymal transition (EMT) and signalling pathway. In vivo studies were also performed to detect the effect of RTN2 on tumour metastasis. We found that RTN2 expression was notably upregulated in tumour tissues compared to pericarcinomatous tissues. High RTN2 expression was positively correlated with patients’ age, vessel invasion, tumour invasion depth, lymph node metastasis and TNM stage. Besides, high RTN2 staining intensity was associated with adverse survival which was further identified as an independent prognostic factor for gastric cancer patients by multivariate analysis. And the predictive accuracy was also improved when incorporated RTN2 into the TNM-staging system. RTN2 could promote the proliferation, migration and invasion of gastric cancer cells in vitro and lung metastasis in vivo. Mechanistically, RTN2 interacted with IP3R, and activated ERK signalling pathway via facilitating Ca^2+^ release from the endoplasmic reticulum, and subsequently drove EMT in gastric cancer cells. These results proposed RTN2 as a novel promotor and potential molecular target for gastric cancer therapies.

## Introduction

Gastric cancer ranks the fifth most frequent and fourth leading cause of cancer-related mortality worldwide [1]. Current surgical treatment combined with chemotherapy and emerging immunotherapy has progressed to improve the survival of gastric cancer patients. However, most patients are diagnosed at an advanced stage and usually occur invasion and metastasis leading to a dismal prognosis [2–5]. Hence, more investigations are urgently needed in unearthing the molecular mechanism of tumour invasion and metastasis, which will be very helpful in determining effective therapies for clinical strategies, and in discovering novel biomarkers to develop specific therapeutic targets for gastric cancer patients.

Reticulon (RTN)/Nogo, which preferred intracellular localisation in the endoplasmic reticulum (ER), was first discovered as a neuroendocrine-specific protein [6]. RTN proteins in mammalian cells can be categorised into four families: reticulon 1, 2, 3 and 4. A conserved C-terminal region termed the reticulon homology domain (RHD), as well as two hydrophobic regions, were existed in all members of the RTN family. Between hydrophobic regions was a Nogo loop composed of 66 amino acids and localised in the ER lumen. The N-terminal domain of each RTN member is unique and may be responsible for the individual functions of each family member [7]. The postulated RTN functions were related to those of the endoplasmic reticulum, including protein processing and secretion, structural stabilisation and maintenance of ER network, ER-associated proapoptotic mechanisms, and transport constituents between ER and other compartments [8]. Previous research about reticulons was focused on neurodegenerative diseases in the past decades [9]. Recently, increasing studies revealed the role of RTNs in cell growth and transmigration in acute injury and multiple kinds of tumours [10–13]. And our previous study delved into the suppressive function of reticulon 3 in hepatocellular carcinoma [14]. However, the expression and biological function of RTNs in human gastric cancer remain little investigated.

In this study, we found that reticulon 2 (RTN2) was upregulated in gastric cancer, and overexpression of RTN2 promotes migration and invasion of tumour cells. RTN2 induced epithelial-to-mesenchymal transition (EMT) via facilitating ERK signalling in an ER Ca^2+^ efflux-dependent manner. Meanwhile, elevated expression of RTN2 was identified as an independent factor that contributed to the poor prognosis in gastric cancer patients.

## Results

### RTN2 expression is upregulated in gastric cancer

To investigate whether RTNs were involved in the progression of gastric cancer, we firstly screened the mRNA expression patterns of the reticulon family in reported TCGA-STAD and two GEO datasets which contained matched gastric cancer specimens. Among the members of the reticulon family, only *RTN2* mRNA level was statistically significantly increased in gastric cancer tissues and displayed a similar tendency in all datasets (Supplementary Fig. 1). Conversely, the alteration of *RTN4* mRNA level was heterogeneous, which was marginally increased in tumour tissues from TCGA-STAD and GSE13861 datasets but downregulated in GES13911 dataset (Supplementary Fig. 1). Although *RTN1* relative mRNA level was decreased in tumour tissues from GES13911 dataset, it was almost unchanged in TCGA-STAD and GSE13861 datasets (Supplementary Fig. 1). *RTN3* mRNA expression was elevated in tumour tissues from the TCGA-STAD and GES13911 datasets, whereas the fold change was relatively low (Supplementary Fig. 1).

**Fig. 1:**
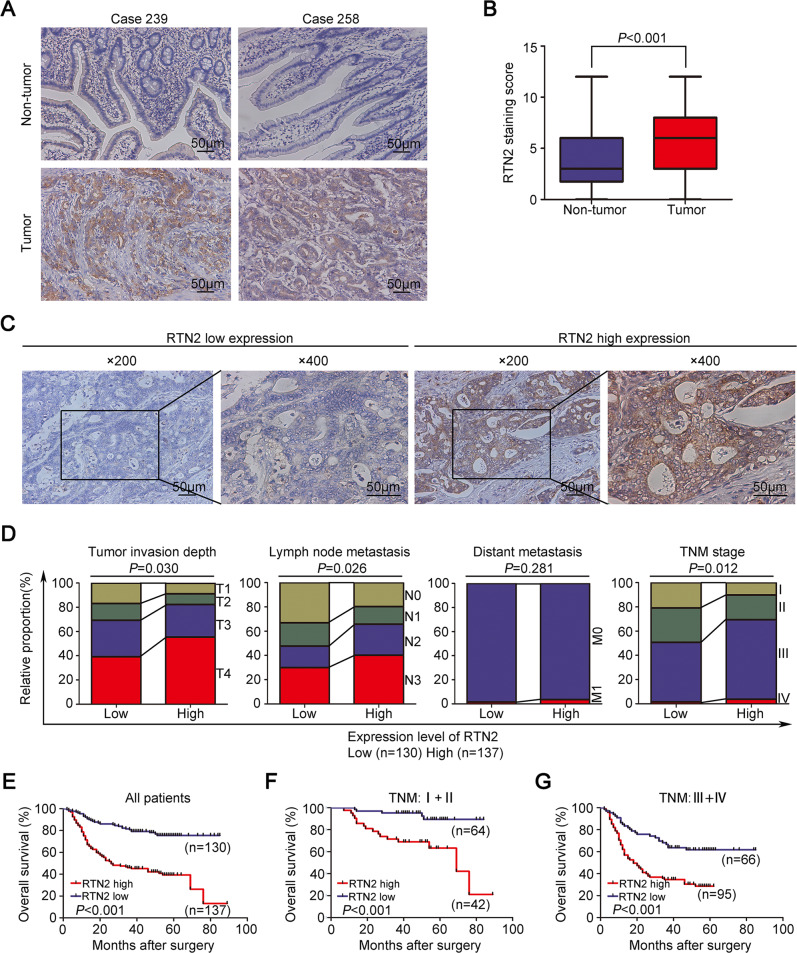
High expression of RTN2 is correlated with tumour progression and adverse prognosis in gastric cancer patients. **A** Representative IHC staining of RTN2 in tumour tissue and matched non-tumour tissue of gastric cancer patients. **B** The comparison of RTN2 staining score in tumour tissue and matched non-tumour tissue of gastric cancer patients. Data are represented as min-to-max bar graphs with median lines. **C** The representative low and high expression of RTN2 in tumour tissues. **D** Correlation of RTN2 expression pattern with tumour invasion depth, lymph node metastasis, distant metastasis, and TNM stage in clinical gastric cancer cases. **E**–**G** Association of RTN2 expression in gastric cancer with overall survival was analysed by Kaplan–Meier survival curve for all patients (**E**), patients at TNM I + II stage (**F**), and patients at TNM III + IV stage (**G**), respectively. Statistical significance was calculated by Student’s two-tailed *t* test (**B**), Pearson χ^2^ test (**D**) and log-rank test (**E**–**G**).

Therefore, a tissue microarray was employed to examine the protein expression of RTN2 in 267 gastric cancer patients with different stages by immunohistochemical analysis. We found that RTN2 was mainly localised in the cell cytoplasm, and the representative staining of RTN2 in tumour and adjacent non-tumour tissues were shown in Fig. 1A. In addition, the expression level of RTN2 in the normal gastric epithelium was relatively low but was obviously upregulated in matched transformed tissues (Fig. 1A). Further statistical analysis demonstrated that the RTN2 staining score in tumour cells was remarkably higher compared to that in the normal gastric epithelium (Fig. 1B).

### Correlations between RTN2 expression and clinicopathological features as well as overall survival in gastric cancer patients

We next evaluated the association between RTN2 expression and clinicopathological features in gastric cancer patients. ROC curve analysis was utilised to estimate the high and low expression of intratumoural RTN2, and representative images were shown in Fig. 1C. Chi-square analysis revealed that high expression of RTN2 was positively correlated with older age (*P* = 0.009), more vessel invasion (*P* = 0.018), tumour invasion depth (*P* = 0.030), lymph node metastasis (*P* = 0.026) and advanced TNM (tumour node metastasis) stage (*P* = 0.012) (Table 1). To better understand the role of RTN2 in the development of gastric cancer, the proportion of different tumour invasion depth, lymph node metastasis, distant metastasis and TNM stage in patients with RTN2 low and high expression are shown in Fig. 1D, respectively. These results reveal that the expression of RTN2 is prone to increase with the progression of gastric cancer.

**Table 1:** Relationship between RTN2 expression and clinicopathological characteristics in patients with gastric cancer.

		RTN2 expression		
		Low	High	
Factors	No.	No. (%)	No. (%)	*P* value
Gender				
Male	184	90 (48.9)	94 (51.1)	0.913
Female	83	40 (48.2)	43 (51.8)	
Age (years)				
<60	126	72 (57.1)	54 (42.9)	**0.009**
≥60	141	58 (41.1)	83 (58.9)	
Tumour size (cm)				
Mean	4.0	3.9	4.1	0.394
Median	3.5	3.0	4.0	
IQR	2.0–6.0	2.0–6.0	2.0–6.0	
Tumour location				
Upper third	33	15 (745.5)	18 (54.5)	0.448
Middle third	59	25 (42.4)	34 (57.6)	
Lower third	175	90 (51.4)	85 (48.6)	
Lauren’s classification				
Intestinal	133	66 (49.6)	67 (50.4)	0.935
Diffuse	125	60 (48.0)	65 (52.0)	
Mixture	9	4 (44.4)	5 (55.6)	
Differentiation				
Poorly differentiated	212	105 (49.5)	107 (50.5)	0.590
Well differentiated	55	25 (45.5)	30 (54.5)	
Vessel invasion				
Absent	196	104 (53.1)	92 (46.9)	**0.018**
Present	71	26 (36.6)	45 (63.4)	
Tumour invasion depth				
T1	34	22 (64.7)	12 (35.3)	**0.030**
T2	30	18 (60.0)	12 (40.0)	
T3	76	39 (51.3)	37 (48.7)	
T4	127	51 (40.2)	76 (59.8)	
Lymph node metastasis				
N0	70	43 (61.4)	27 (38.6)	**0.026**
N1	45	25 (55.6)	20 (44.4)	
N2	58	23 (39.7)	35 (60.3)	
N3	94	39 (41.5)	55 (58.5)	
Distant metastasis				
Absent	260	128 (49.2)	132 (50.8)	0.281
Present	7	2 (28.6)	5 (71.4)	
TNM stage				
I	41	27 (65.9)	14 (34.1)	**0.012**
II	65	37 (56.9)	28 (43.1)	
III	154	64 (41.6)	90 (58.4)	
IV	7	2 (28.6)	5 (71.4)	

*TNM* tumour node metastasis, IQR interquartile range.

*P* value < 0.05 marked in bold font shows statistical significance.

Then Kaplan–Meier analysis was used to evaluate the correlation between intratumoural RTN2 expression and overall survival of gastric cancer patients after gastrectomy. And results demonstrated that the overall survival of RTN2 high expression group was shorter than that in RTN2 low expression group (Fig. 1E). The overall survival rate of patients with RTN2 low expression exhibited nearly two times higher than those with RTN2 high expression (76.9% vs 40.1%). To further examine whether RTN2 expression could stratify patients with different TNM stage, we grouped the TNM I + II and TNM III + IV as early and advanced-stage diseases, respectively. Similarly, high expression of RTN2 was correlated with depressed overall survival in both subgroups of gastric cancer patients (Fig. 1F, G).

### RTN2 expression is an independent prognostic factor for gastric cancer patients

**Fig. 2:**
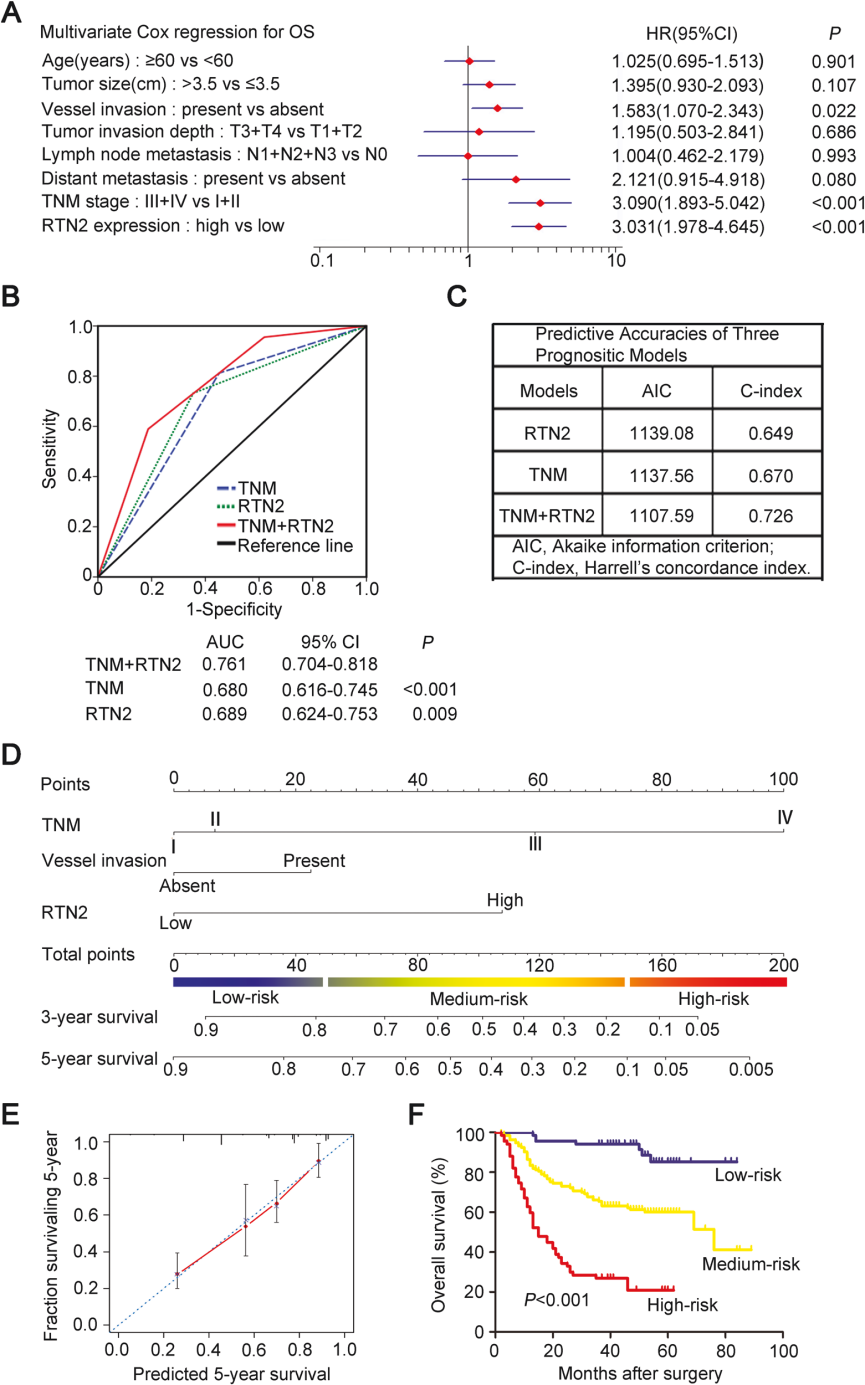
RTN2 expression is an independent factor that could improve predictive accuracy in gastric cancer patients. **A** Multivariate Cox analysis was performed to identify independent prognostic factors in patients with gastric cancer. **B** ROC analysis of the sensitivity and specificity for the predictive value of TNM model, RTN2 expression model and the combined model. **C** The predictive accuracies of TNM staging, RTN2 expression and the combined model were compared by AIC and C-index. **D** Nomogram was utilised to quantify the integrated effect of the proven independent prognostic factors for overall survival. **E** Calibration plot of the nomogram for 5-year survival. **F** Of all 267 patients, three groups were divided according to the total points in the nomogram which range of 0–50, 51–150, 151–200, was defined as low-, medium- and high-risk subgroup, respectively. Kaplan–Meier analysis was used to test the correlation of the risk with overall survival. Statistical significance was calculated by multivariate Cox analysis (**A**) and log-rank test (**F**).

To identify the prognostic factors for overall survival in gastric cancer patients, univariate and multivariate analyses were performed. In the univariate analysis, patients’ age (HR, 1.515; 95% CI, 1.043–2.202; *P* = 0.029), tumour size (HR, 1.891; 95% CI, 1.301–2.749; *P* = 0.001), vessel invasion (HR, 2.580; 95% CI, 1.639–4.060; *P* < 0.001), tumour invasion depth (HR, 2.357; 95% CI, 1.555–3.571; *P* < 0.001), lymph node metastasis (HR, 2.396; 95% CI, 1.598–3.591; *P* < 0.001), distant metastasis (HR, 10.20; 95% CI, 2.353–44.17; *P* = 0.002), TNM stage (HR, 3.234; 95% CI, 2.222–4.707; *P* < 0.001), and RTN2 expression (HR, 3.401; 95% CI, 2.330–4.964; *P* < 0.001) were identified as risk features that might affect gastric cancer patients’ overall survival. (Supplementary Table 1). Further assess based on multivariate Cox regression analyses illustrated that tumour vessel invasion (HR, 1.583; 95% CI, 1.070–2.343; *P* = 0.022), TNM stage (HR, 3.090; 95% CI, 1.893–5.042; *P* < 0.001), and RTN2 expression (HR, 3.031; 95% CI, 1.978–4.645; *P* < 0.001) had independent prognostic significance for overall survival of gastric cancer patients (Fig. 2A).

Next, we combined RTN2 expression with TNM-staging system to construct a more accurate predictive model for outcomes of gastric cancer patients. ROC analysis was performed to compare the prognostic sensitivity and specificity integrated RTN2 expression with TNM-staging system and each of them alone. As shown in Fig. 2B, the incorporation of RTN2 expression and TNM-staging system exhibited a prominent higher prognostic value (AUC, 0.761; 95% CI, 0.704–0.818) compared to RTN2 expression alone (AUC, 0.689; 95% CI, 0.624–0.753; *P* = 0.009), or TNM staging alone (AUC, 0.680; 95% CI, 0.616–0.745; *P* < 0.001). The AIC was significantly reduced (1137.56 vs 1107.59), and the C-index was obviously increased (0.670 vs 0.726) when the predictive model was established by uniting TNM-staging system and RTN2 expression than the former alone (Fig. 2C). Taken together, incorporation TNM staging system and RTN2 expression could create a more mightily predictive model for the overall survival of patients with gastric cancer.

### Predictive nomogram for overall survival

To provide a quantitative method for better outcome prediction, a nomogram was constructed. In the nomogram, all proven independent prognostic factors including vessel invasion, TNM stage and RTN2 expression were integrated (Fig. 2D). A higher total point indicates a poorer overall survival in this nomogram, and the predicted 3-year and 5-year survival was shown in our results. The calibration plot for this nomogram predicting 5-year survival executed well with the ideal model (Fig. 2E). Then we divided the patients into three groups by the total points, low-risk, medium-risk and high-risk subgroup, and the prognostic significance for overall survival was remarkably (*P* < 0.001) (Fig. 2F), suggesting that this model might refine survival prediction for gastric cancer patients after surgery.

### RTN2 promotes migration and invasion of gastric cancer cells in vitro

**Fig. 3:**
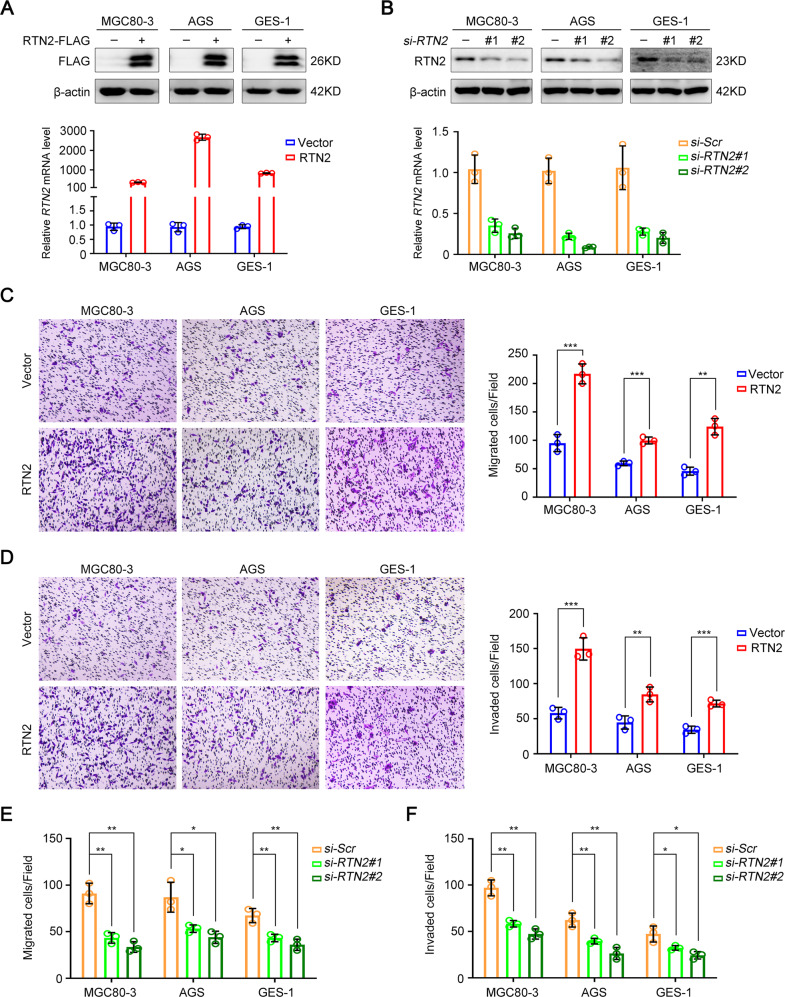
RTN2 promotes migration and invasion of gastric cancer cells in vitro. **A**, **B** The overexpression (**A**) and knockdown (**B**) efficiencies of RTN2 in MGC80-3, AGS and GES-1 cells were monitored by western blot (upper panel) and real-time PCR (lower panel), respectively. **C**–**F** Transwell assays were employed to determine the influence of RTN2 on the migratory and invasive abilities in MGC80-3, AGS and GES-1 cells. **C**, **D** The effects of RTN2 overexpression on the migratory (**C**) and invasive (**D**) abilities in MGC80-3, AGS and GES-1 cells. **E**, **F** The effects of *RTN2* knockdown on the migratory (**E**) and invasive (**F**) abilities in MGC80-3, AGS and GES-1 cells. Data are represented as mean ± SD (**A**–**F**), and statistical significance was calculated by Student’s two-tailed *t* test (**C**–**F**). *P < 0.05; **P < 0.01; ***P < 0.001.

We next investigated the potential function of RTN2 in tumour characteristics in human normal gastric epithelial cell line GES-1 and two kinds of human gastric cancer cell lines including MGC80-3 and AGS (Fig. 3A, B). As RTN2 expression was related to tumour invasion depth in gastric cancer patients, the effects on cell migration and invasion were investigated. Transwell assays demonstrated that both gastric cancer cells exhibited a higher migratory and invasive potential when RTN2 was overexpressed (Fig. 3C, D), while the migratory and invasive abilities were impaired when *RTN2* was knocked down (Fig. 3E, F). Similar to gastric cancer cells, the number of migratory and invasive GES-1 cells were increased or reduced along with RTN2 overexpression or depletion, respectively (Fig. 3C–F). Furthermore, RTN2 overexpression significantly facilitated the viability and colony-formation ability of gastric cancer cells, while *RTN2* knockdown exhibited opposite effects (Supplementary Fig. 2). These results imply that RTN2 might play a stimulative role in cellular processes for metastasis of gastric cancer cells, as well as in the malignant transformation of stomach epithelial cells. Though one of the postulated RTN functions might relate to proapoptotic mechanisms [8], overexpression or knockdown of RTN2 showed little effect on cellular apoptosis in all these cells (Supplementary Fig. 3).

### RTN2 promotes metastasis of gastric cancer cells in vivo

**Fig. 4:**
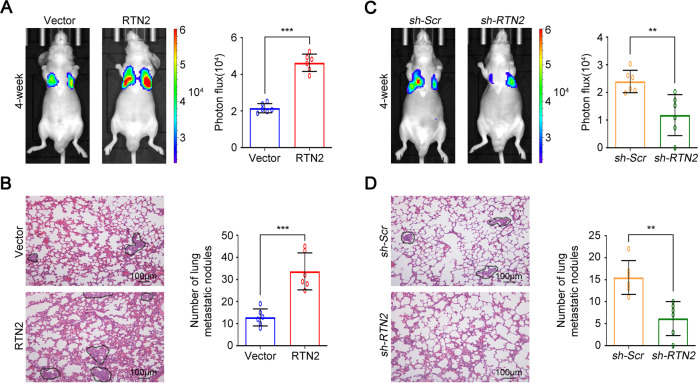
RTN2 promotes metastasis of gastric cancer cells in vivo. Stable MGC80-3 cells were injected into the lateral tail vein of mice as described in the “Materials and Methods”, and photon fluxes were monitored 4 weeks later. **A**, **B** The effects of RTN2 overexpression on metastasis of gastric cancer cells. **A** The luminescent tumour signals were measured (right panel, *n* = 6), and representative images were shown (left panel). **B** Representative hematoxylin-eosin staining of the lung for each group (left panel), micrometastatic lesions were indicated with a dotted line. Numbers of lung metastatic foci in each group were counted (right panel). **C**, **D** The effects of *RTN2* knockdown on metastasis of gastric cancer cells. **D** The luminescent tumour signals were measured (right panel, *n* = 6), and representative images were shown (left panel). **D** Representative hematoxylin-eosin staining of the lung for each group (left panel), micrometastatic lesions were indicated with a dotted line. And numbers of lung metastatic foci in each group were counted (right panel). In (**A**–**D**), data are represented as mean ± SD, and statistical significance was calculated by Student’s two-tailed *t* test. **P < 0.01; ***P < 0.001.

We next injected stable MGC80-3-luciferase cells into the lateral tail vein of BALB/C nude mice and monitored tumour metastasis in vivo. As shown in Fig. 4A, RTN2 overexpression remarkably enhanced luminescent tumour signal in nude mice. After the sacrifice of mice, we found that more and larger micrometastatic lesions were detected in the lungs of nude mice bearing with RTN2-transfected cells (Fig. 4B). Nevertheless, nude mice inoculated with *RTN2* shRNA-transfected MGC80-3-luciferase cells exhibited weak luminescent tumour signal compared with those inoculated with control cells (Fig. 4C). Meanwhile, knockdown of *RTN2* notably restrained the formation of microtumour lesions and metastatic nodules in the lungs (Fig. 4D). Furthermore, we also utilised stable RTN2 overexpression or knockdown gastric cancer cells to establish an animal model of peritoneal metastasis. As shown in Supplementary Fig. 4A, the number of metastatic nodules was significantly increased compared RTN2 overexpression group with the control group. Conversely, RTN2 depletion impaired the peritoneal metastatic ability of gastric cancer cells (Supplementary Fig. 4B). These data indicate that RTN2 might facilitate metastasis of gastric cancer cells in vivo.

### RTN2 facilitates epithelial-to-mesenchymal transition in gastric cancer cells

**Fig. 5:**
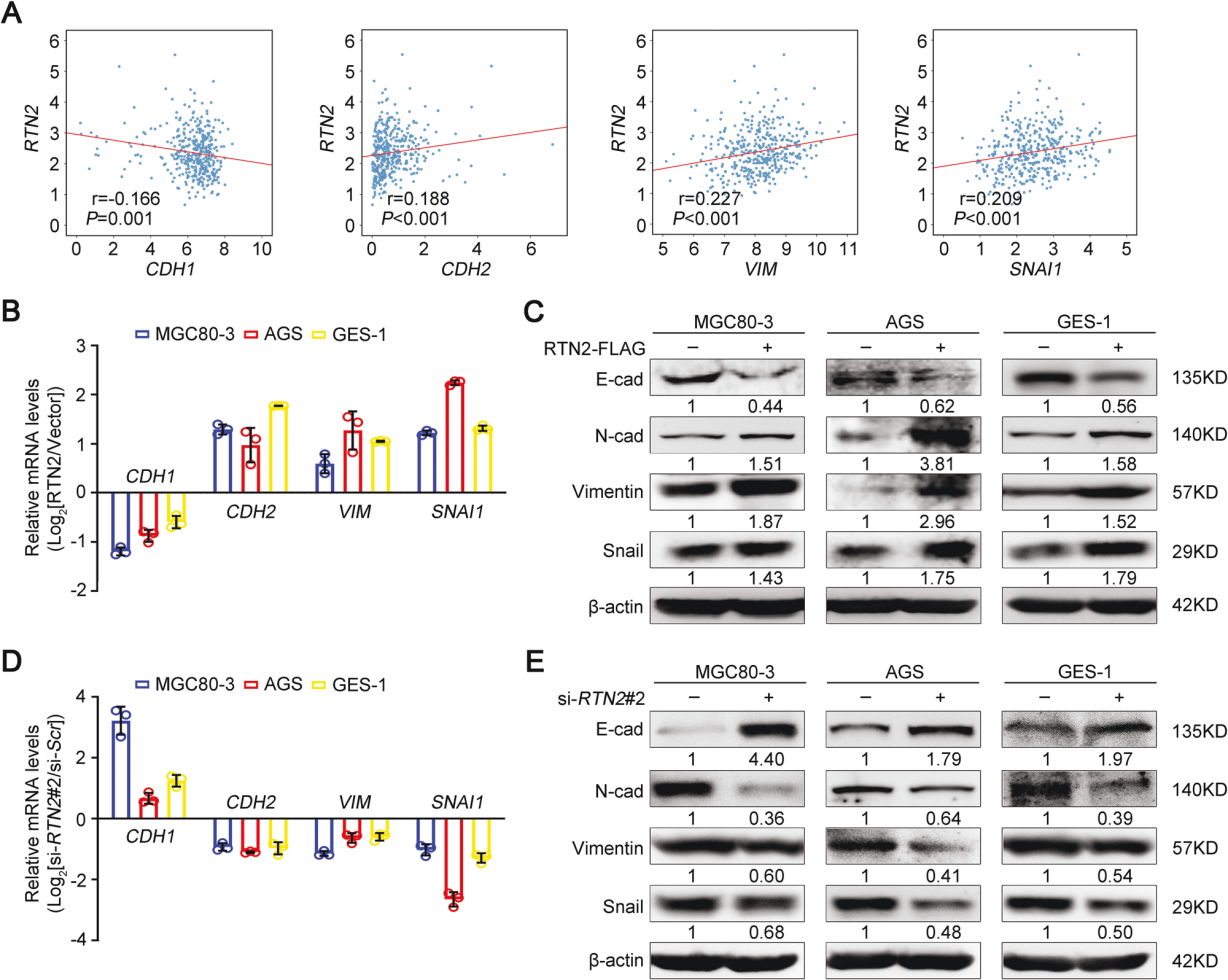
RTN2 facilitates epithelial-mesenchymal transition of gastric cancer cells. **A** Spearman’s r test was utilised to evaluate the correlation between *RTN2* mRNA expression and EMT markers *CDH1*, *CDH2*, *VIM*, *SNAI1* in TCGA-STAD dataset, respectively. **B**, **C** The effects of RTN2 overexpression on EMT markers in MGC80-3 and AGS were examined by real-time PCR (**B**) and western blot (**C**), respectively. **D**, **E** The effects of *RTN2* knockdown on EMT markers in MGC80-3 and AGS were assessed by real-time PCR (**D**) and western blot (**E**), respectively. **B**, **D** Data are represented as mean ± SD. Statistical significance was calculated by Spearman’s *r* test (**A**).

Epithelial-to-mesenchymal transition (EMT) enables tumour cells with motile and invasive properties in tumour progression [15, 16]. We next explored the potential correlation between *RTN2* mRNA expression and EMT markers in the TCGA-STAD dataset. As shown in Fig. 5A, *RTN2* mRNA expression was negatively correlated with the epithelial marker E-cadherin (*CDH1*), whereas it was positively correlated with the mesenchymal marker N-cadherin (*CDH2*) and Vimentin (*VIM*) as well as transcriptional repressor Snail (*SNAI1*). Real-time PCR analysis showed that in RTN2-overexpressed MGC80-3, AGS and GES-1 cells, the mRNA level of *CDH1* was reduced along with an upregulated expression of *CDH2*, *VIM* and *SNAI1* (Fig. 5B). Similar effects on protein expression of EMT markers were also observed in RTN2-overexpressed cells (Fig. 5C). However, opposite effects were obtained in RTN2-depleted cells (Fig. 5D, E). Besides, we also examined the effect of RTN2 on focal adhesion by staining paxillin, a focal adhesion-associated adaptor protein. Confocal imaging displayed that more and larger paxillin clusters were detected in RTN2-overexpressed gastric cancer cells (Supplementary Fig. 5A). Conversely, *RTN2* knockdown attenuated both the number and the size of paxillin clusters (Supplementary Fig. 5B). Together, these results suggest that RTN2 enhances EMT and the development of focal adhesion in gastric cancer cells.

### RTN2 contributes to tumour migration and invasion through ER Ca^2+^ efflux-induced ERK activation

**Fig. 6:**
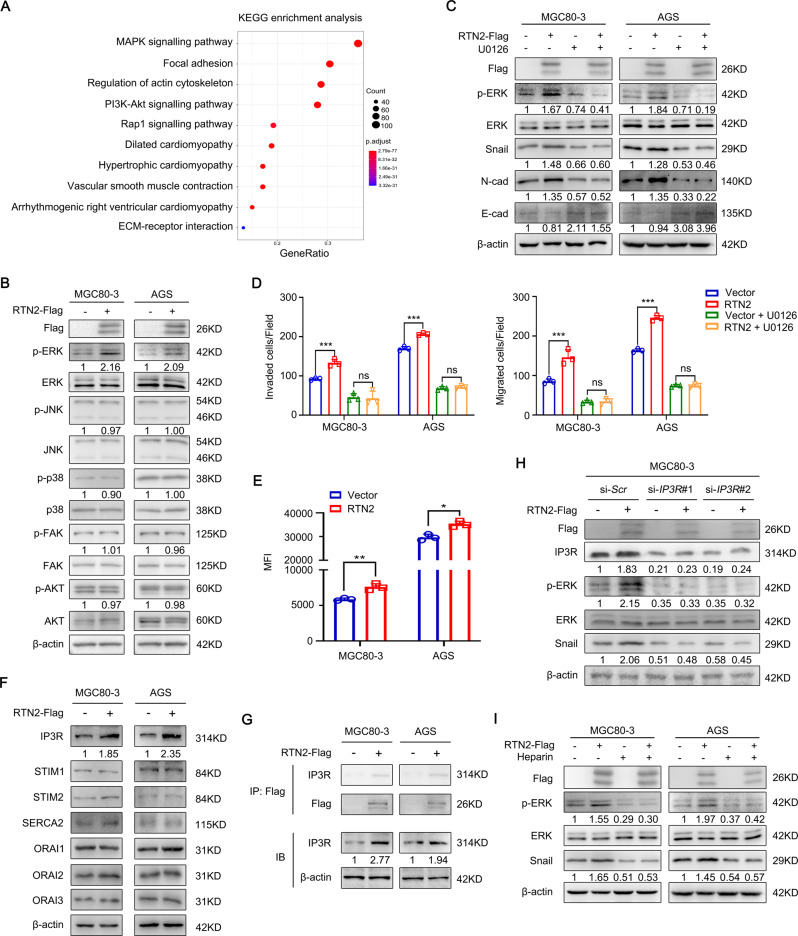
RTN2 promotes EMT, migration and invasion of gastric cancer cells through ER Ca^2+^ efflux-mediated ERK activation. **A** KEGG pathway enrichment analysis of RTN2-associated signalling pathways. **B** Western blot validation of predicted pathways. **C** The effects of U0126 (10 μM) on RTN2-associated ERK phosphorylation and EMT markers. **D** Transwell assays were performed to determine the invasion (left panel) and migration (right panel) capacities of MGC80-3 and AGS cells. **E** Cytoplasmic Ca^2+^ was evaluated by FACS after staining with Fluo-3 AM in RTN2-overexpressed or controlled gastric cancer cells. **F** The effects of RTN2 on Ca^2+^-related channels including IP3R, SERCA2, STIM and ORAI were examined by western blot. **G** MGC80-3 and AGS cells were transfected with RTN2 or empty vector, and applied to immunoprecipitation assay. **H** Gastric cancer cells were transfected as indicated and applied to western blot. **I** MGC80-3 and AGS were transfected with RTN2 or empty vector, treated with or without heparin (5 mg/mL), and then applied to western blot. **D**, **E** Results are presented as mean ± SD. Statistical significance was calculated by Student’s two-tailed *t* test. *P < 0.05; **P < 0.01; ***P < 0.001; ns no significance.

To further explore the molecular mechanism responsible for the accelerative function of RTN2 in gastric cancer metastasis, we first performed KEGG pathway enrichment analysis to identify potential signalling pathways associated with RTN2 using the TCGA-STAD database (Fig. 6A). Results indicated that RTN2 high expression was positively related to the MAPK signalling pathway, focal adhesion and PI3K-AKT signalling pathway (Fig. 6A). However, further study revealed that RTN2 overexpression facilitated the phosphorylation of ERK but not JNK or p38 in MAPK pathway, and RTN2 also showed little effect on FAK and AKT which was related to focal adhesion and PI3K-AKT signalling pathway, respectively (Fig. 6B). Then MEK1/2 inhibitor U0126 which could block ERK phosphorylation was used to verify the role of the ERK pathway in the pro-metastatic effects of RTN2. As shown in Fig. 6C, ERK inhibition abrogated RTN2-mediated upregulation in the protein level of Snail and N-cadherin as well as the reduction of E-cadherin. Besides, the promotive influence of RTN2 on the migratory and invasive capacities was also blocked by U0126 (Fig. 6D). Hence, these findings imply that the pro-metastatic impacts of RTN2 in gastric cancer is ERK-dependent.

Given previous studies that ER-resident reticulons were closely related to ER Ca^2+^ flux, and intracellular Ca^2+^ was critical for the activation of ERK [14, 17], we evaluated the effect of RTN2 on ER Ca^2+^ release in gastric cancer cells. Mag-Fluo-4 AM was applied to examine the Ca^2+^ content in ER. Fluorescence imaging showed that the Ca^2+^ concentration in ER was obviously reduced when RTN2 was overexpressed, while was augmented by RTN2 depletion (Supplementary Fig. 6A). Meanwhile, flow cytometry analysis of Fluo-3 AM indicated that RTN2 overexpression enhanced the Ca^2+^ efflux from ER (Fig. 6E). In addition, we found that ER Ca^2+^ efflux-related channels IP3R was upregulated following RTN2 overexpression, but was repressed by *RTN2* knockdown (Fig. 6F and Supplementary Fig. 6B–D). However, the effects of RTN2 on other channels including SERCA2, STIM and ORAI were marginal or inconsistent between the two gastric cancer cells (Fig. 6F and Supplementary Fig. 6B–D). Furthermore, immunoprecipitation analysis exhibited that IP3R was associated with RTN2, and knockdown of *IP3R* blocked RTN2-mediated the phosphorylation of ERK and the expression of Snail (Fig. 6G, H and Supplementary Fig. 6E). Taken together, these data suggest that IP3R probably be the target molecule of RTN2 interaction.

Next, heparin was administrated to inhibit Ca^2+^ release from ER to cytosol, which resulted in the abolishment of ERK phosphorylation and Snail expression increased by RTN2 overexpression (Fig. 6I). Furthermore, BAPTA-AM was used as a cell-permeant chelator of Ca^2+^ store to examine the involvement of cytosolic calcium in EMT and migration. The results indicated that blockade of cytosolic calcium by BAPTA-AM reversed RTN2-induced ERK/Snail signalling activation as well as the migration of gastric cancer cells (Supplementary Fig. 7). Therefore, RTN2 could promote ER Ca^2+^ efflux through upregulating IP3R, leading to the ERK activation as well as tumour migration and invasion of gastric cancer cells.

## Discussion

The traditional predictive model for outcomes of patients with gastric cancer mainly depends on TNM-staging system including the information derived from tumour cell invasion depth, lymph node metastasis and distant metastasis. However, its ability to distinguish a subgroup of patients is limited due to the heterogeneity of tumour. Therefore, identifying new molecules associated with tumorigenesis in tumour cells would be beneficial in understanding the development of gastric cancer. Reticulons have been widely studied on the nervous system since the discovery of its rich expression in brain tissue [18–21]. Among the various members of the RTN family, RTN4B was most extensively studied as it was expressed ubiquitously. In this study, we analysed RTNs expression in paired gastric cancer samples from different reported datasets and found that RTN2 mRNA level was coincidently upregulated in tumour tissues compared with non-tumour tissues (Supplementary Fig. 1). It implied that RTN2 might play an important role in the development of gastric cancer. In vivo and in vitro studies also indicated that RTN2 promoted the proliferation, epithelial-to-mesenchymal transition and metastatic potential in gastric cancer cells (Figs. 3–5 and Supplementary Fig. 2).

Accumulating studies have demonstrated the involvement of RTNs in several other kinds of diseases except for neuropathy. Knockdown of RTN1A and RTN4B would attenuate renal and liver fibrosis, respectively [22, 23]. Nevertheless, RTN4B facilitates alcoholic liver disease through regulation of Kupffer cell polarisation [24]. And more remarkably, dysregulation of RTNs in multiple tumours has also been investigated. Expression of RTN4A/B was downregulated in intrahepatic cholangiocarcinoma, malignant melanoma and non-small cell lung carcinomas [25–27]. And all these evidence suggest that the expression and function of RTNs may not be limited to nervous tissue. In this study, we found that RTN2 expression was increased in gastric cancer (Fig. 1A, B and Supplementary Fig. 1). And IHC data clarified that upregulation of RTN2 was positively associated with tumour progression and could be regarded as a potential unfavourable prognostic marker for gastric cancer patients. And RTN2 expression could refine the risk stratification system which was based on the TNM stage alone (Fig. 2B, C). However, the study was retrospectively designed in nature. All these results need a larger, multicenter, prospective dataset to validate. Besides, though the dysregulation of RTNs in cancers has drawn more attention nowadays, how the expression of RTNs is influenced remains little understood. A previous study revealed a likely mechanism of RTN4A downregulation via the degradation through the ubiquitin–proteasome pathway [28]. Nevertheless, our study demonstrated that the mRNA levels of RTN2 were increased in gastric cancer, suggesting that RTN2 might be transcriptionally upregulated during gastric tumorigenesis.

As endoplasmic reticulum resident proteins, RTNs were necessary for endoplasmic reticulum tubulation, intracellular trafficking and calcium flowing [29, 30]. These functions were related to the transformation of cell morphology, ER stress and apoptosis. In addition to the effects on the growth of tumour cells [31], RTNs have been reported to be involved in regulating cell migration and invasion. According to previous studies, RTN4A may inhibit the migration and invasion of human malignant glioma cells via the downregulation of RhoA-cofilin signalling [12]. However, its isoform RTN4B promotes the epithelial-mesenchymal transition of HeLa cervical cancer cells via Fibulin-5, an extracellular matrix protein [32]. Besides, RTN3 stimulated primordial germ cell migration through interaction with, and regulation of, CXCR4 [33]. Therefore, the members of the RTN family may play dual roles in regulating cell migration/invasion. Another study also indicated that Nogo-66, an inhibitory domain of RTN4A, mediates glycogen synthase kinase-3β (GSK-3β) activation in mouse neuroblastoma cells [34]. GSK-3β might contribute to the modulation of several downstream signalling pathways such as β-catenin/Snail/E-cadherin, which have been identified to regulate EMT, metastasis and the progression of various cancers [35–37]. In this study, our results demonstrated that RTN2 promotes proliferation, migration, invasion and EMT in gastric cancer cells in vitro through ER Ca^2+^ efflux-mediated ERK activation (Figs. 3, 5, 6 and Supplementary Fig. 2). Furthermore, RTN2 could also accelerate metastasis of gastric cancer cells in vivo (Fig. 4), implying that the enhanced proliferation of gastric cancer cell benefits for its survival in circulation, extravasation efficiency and the efficiency of tumour cells outgrowth in the lung. In addition, we found that RTN2 could interact with IP3R, one of the intracellular Ca^2+^ release channels, for the first time (Fig. 6G). IP3R was shown to either suppress cancer by promoting cellular senescence or support cancer by driving metabolism, proliferation, invasion and so on [38, 39]. However, the precise role of IP3R in gastric cancer remains unclear. Our present results might provide clues towards a better understanding of the pro-tumour functions of IP3R in gastric cancer, which could be promoted by RTN2 to stimulate downstream ERK signalling.

In conclusion, our work revealed that high RTN2 expression was an independent and adverse predictor of overall survival in gastric cancer patients. A more accurate predictive model for outcomes could be established by combining RTN2 expression and the TNM-staging system. RTN2 promoted ER Ca^2+^ release, and subsequently activated the ERK signalling pathway which droved EMT and led to the metastasis of gastric cancer cells. Thus, RTN2 might represent a new biomarker for gastric cancer prognosis and targeting RTN2 may provide a novel approach for the treatment of gastric cancer.

## Materials and methods

### Patient samples

All of the methods were approved by the research medical ethics committee of Zhongshan Hospital and were performed in accordance with the approved guidelines. Tumour and matched peritumoral specimens were obtained from 267 gastric cancer patients who underwent surgical resection without preoperative treatment from 2004 to 2008, at the Department of General Surgery, Zhongshan Hospital (Fudan University, Shanghai, China). The diagnosis of gastric cancer was confirmed by pathologic examination. Patients’ clinicopathological characteristics, date of surgery, tumour stage, Lauren’s type, tumour location, surgical treatment methods, survival time, and other relevant clinicopathological data were obtained from hospital records. The use of human tissue samples and clinical data were approved by the Research Ethics Committee of Zhongshan Hospital (B2021-711). Informed consent was obtained from all patients.

### Tissue microarray construction and immunohistochemistry (IHC)

Tissue microarray construction was carried out as previously described [40]. In brief, the tissue microarrays were baked at 60 °C for 6 hours, dewaxed in xylene, rehydrated through a gradient concentration and blocked the endogenous peroxidase activity by 3% hydrogen peroxide. After antigen retrieving by citrate buffer using a microwave oven, the sections were incubated with the primary antibody RTN2 (purchased from Proteintech, Chicago, IL, USA, 1:100 dilution) at 4 °C overnight. Then, tissue sections were treated with Primary Antibody Amplifier Quanto and HRP Polymer Quanto (Thermo Scientific, Fremont, CA, USA). Finally, the sections were visualised by DAB solution and counterstained with haematoxylin. IHC staining score was assessed by two independent pathologists who were blinded to the patients’ clinicopathological data. The score for the extent of the IHC-stained area was set as 0 for <5%; 1 for 5–25%; 2 for 26–50%; 3 for 51–75%; and 4 for 76–100% of tumour cells stained. The score for IHC intensity was also scaled as 0 for no IHC signal, 1 for weak, 2 for moderate and 3 for strong. The final score used in the analysis was calculated by multiplying the extent score and intensity score. Values less than or equal to 4 were considered as the low expression, based on receiver operating characteristic (ROC) analysis.

### Cell culture

Human gastric cancer cell lines MGC80-3, AGS and normal epithelial cell line GES-1 were obtained from the Shanghai Cell Bank of Chinese Academy of Sciences (Shanghai, China). All cell lines were cultured in Dulbecco’s modified Eagle’s medium replenished with 10% FBS (Gibco, Grand Island, NY, USA), and cultured at 37 °C in a humidified 5% CO_2_ incubator. MGC80-3 and AGS cells stably expressing RTN2 or sh-*RTN2* were generated by infecting with corresponding lentiviral particles, and clones were selected with puromycin (0.5 µg/mL for MGC80-3 and 1 µg/mL for AGS).

### Plasmids construction, short hairpin RNA (shRNA), small interfering RNA (siRNA) synthesis and transfections

The coding sequence of human RTN2 was amplified by PCR and constructed into the p3×FLAG-CMV-14 vector (Sigma, St. Louis, MO, USA) or LV17 (EF-1a/Luciferase17&Puro) vector (Gene Pharma, Shanghai, China). The shRNA specifically targeting *RTN2* (AGCTAAGATCCGAGCTAAA) and scrambled control shRNA (TTCTCCGAACGTGTCACGT) were synthesised by Gene Pharma and constructed into the LV16 (U6/Luciferase17&Puro) vector. The siRNA specifically targeting *RTN2* (si-*RTN2*#1: CCGUCUACAGCCUCCUCAA; si-*RTN2*#2: AGCUAAGAUCCGAGCUAAA), *IP3R* (si-*IP3R*#1: AUGCCCUUACAUCUGAACCU; si-*IP3R*#2: GUACCCUCAACCGGUGCAAA) and scrambled control siRNA (UUCUCCGAACGUGUCACGUTT) were synthesised by Biotend (Shanghai, China). Cells were transfected with the plasmids or siRNAs using Lipofectamine 2000 (Invitrogen, Carlsbad, CA, USA) following the manufacturer’s instructions.

### Transwell assays

Gastric cancer cells MGC80-3 and AGS were transfected as indicated. Transwell migration and invasion assays were performed in 12-well transwell plates (8-μm pore size) according to the manufacturer’s instructions (Millipore, Cambridge, MA, USA). For invasion assays only, the bottom of the transwell chamber was coated with BD Matrigel Basement Membrane Matrix (BD Biosciences, San Diego, CA, USA). In all, 1 × 10^5^ cells in basic culture medium without serum were added into the upper chamber, and the lower chamber was filled with culture medium containing 20% FBS as a chemo-attractant. Migration and invasion of cells were determined 24 hours and 48 hours later, respectively. Cells on the upper side of the chamber were removed from the surface of the membrane by scrubbing, and cells on the lower surface of the membrane were fixed with 4% paraformaldehyde and stained with 0.1% crystal violet. The number of infiltrating cells was counted in three randomly selected microscopic fields of each filter.

### Colony-formation assay

Briefly, 4000 stable RTN2 overexpression or knockdown or the corresponding control MGC80-3 and AGS cells were seeded in 12-well plates and cultured at 37 °C under 5% CO_2_. After 2 weeks, colonies were fixed with 4% paraformaldehyde and stained with crystal violet. Experiments were performed in triplicate.

### Cell counting Kit-8 (CCK-8) assay

The proliferation of gastric cancer cells was measured by the CCK-8 assay. The stable RTN2 overexpression or knockdown or the corresponding control cells were seeded into 96-well plates (1000 cells per well) and cultured for 24, 48, 72 and 96 hours. Then incubated cells with CCK-8 solution for 1 hour. The absorbance at 450 nm was gathered by a microplate spectrophotometer.

### Animal studies

Four to six-week-old male BALB/C nude mice were purchased from Shanghai Laboratory Animal Center of Chinese Academy Sciences, housed in a specific pathogen-free room, and grouped randomly. Animal care and experiments were performed in strict accordance with the “Guide for the Care and Use of Laboratory Animals” prepared by the National Academy of Sciences and published by the National Institutes of Health, and all animal experiments were approved by the ethics committee of Fudan University. For RTN2 overexpression, equal mass of empty vector or RTN2 plasmids were transfected into MGC80-3 cells. For *RTN2* knockdown, equal mass of scrambled control shRNA or *RTN2* shRNA were transfected into MGC80-3 cells. Forty-eight hours later, transfected cells were collected, and resuspended in phosphate-buffered saline at the concentration of 2.5 × 10^7^/mL. For the mouse model of lung metastasis, each mouse was primed with 200 µL cell suspension by tail intravenous injection. Four weeks later, all mice were fluorescence imaged by the IVIS Spectrum CT (PerkinElmer, Waltham, MA, USA) after intraperitoneal injection with 3 mg d-luciferin potassium in 200 µL PBS. Then, they were sacrificed and lung tissues were taken out and fixed in paraformaldehyde solution to prepare tissue sections. For the mouse model of peritoneal metastasis, each mouse was primed with 200 µL cell suspension by intraperitoneal injection. Two weeks later, all mice were sacrificed and the number of peritoneal metastatic nodules were counted.

### Western blot

Briefly, polyacrylamide gel electrophoresis was used to separate proteins which extracted from cells, and transferred onto polyvinylidene fluoride membranes. Membranes were incubated with primary antibodies including: FLAG (1:2000; Sigma**)**, E-cadherin (1:500; Santa Cruz, Dallas, TX, USA), N-cadherin (1:500; Santa Cruz), Vimentin (1:1000; Cell Signalling Technology, Beverly, MA, USA), Snail (1:1000; Cell Signalling Technology), JNK (1:1000; Cell Signalling Technology), phospho-JNK (1:500; Cell Signalling Technology), p38 (1:1000; Cell Signalling Technology), phospho-p38 (1:500; Cell Signalling Technology), AKT (1:1000; Cell Signalling Technology), phospho-AKT (1:500; Cell Signalling Technology), FAK (1:1000; Cell Signalling Technology), phospho-FAK (1:500; Cell Signalling Technology), ERK (1:1000; ABclonal Biotech Co., Ltd., Cambridge, MA, USA), phospho-ERK (1:500; Abclonal), IP3R (1:1000; Santa Cruz), STIM1 (1:500; Santa Cruz), STIM2 (1:500; Santa Cruz), SERCA2 (1:500; Santa Cruz), ORAI1 (1:500; Santa Cruz), ORAI2 (1:500; Santa Cruz), ORAI3 (1:500; Santa Cruz), β-actin (1:3000; Proteintech) and then with HRP-conjugated secondary antibody. At last, an enhanced chemiluminescence assay was used to detect the reactions. All original western blots were provided as supplementary materials.

### Immunoprecipitation

Cells were transfected as indicated, and then were collected by immunoprecipitation lysis buffer (Beyotime, Shanghai, China). Next equal amounts of cell lysis were incubated with FLAG antibody (1:200; Sigma) immobilised onto Protein G-Sepharose beads for 6 hours at 4 °C with gentle rotation. Then the beads were washed by lysis buffer three times. After adding with SDS-PAGE sample loading buffer and subsequently boiling, the beads were centrifuged to acquire supernatant for western blot.

### Real-time PCR

Total RNA was purified from gastric cancer cells using TRIzol (Invitrogen) according to the manufacturer’s instructions. The RNA was then processed for reverse transcription and quantitative PCR using a Takara RNA PCR Kit and SYBR Premix Ex Taq (Takara, Tokyo, Japan) in accordance with the manufacturer’s instructions. GAPDH was used as an internal control. The primers used were as follows: *RTN2* sense: CTTTAGCATCGTGTCCGTGG, anti-sense: CTTTGCGGTAAACCCTGAGAG; *CDH1* sense: TACACTGCCCAGGAGCCAGA, anti-sense: TGGCACCAGTGTCCGGATTA; *CDH2* sense: CAGTATCCGGTCCGATCTGC, anti-sense: GTCCTGCTCACCACCACTAC; *VIM* sense: GGACCAGCTAACCAACGACA, anti-sense: AAGGTCAAGACGTGCCAGAG; *SNAI1* sense: TCTGAGGCCAAGGATCTCCA, anti-sense: TGGCTTCGGATGTGCATCTT; *GAPDH*, sense: GAGTCAACGGATTTGGTCGT, anti-sense: TTGATTTTGGAGGGATCTCG; *IP3R* sense: GTGACAGGAAACATGCAGACTCG, anti-sense: CAGCAGTTGCACAAAGACAGGC; *STIM1* sense: CACTCTTTGGCACCTTCCACGT, anti-sense: CTGTCACCTCGCTCAGTGCTTG; *STIM2* sense: CAGTCTTTGGGACTCTGCACGT, anti-sense: GCCAGCGAAAAAGTCGTTCTCG; *SERCA2* sense: GGACTTTGAAGGCGTGGATTGTG, anti-sense: CTCAGCAAGGACTGGTTTTCGG; *ORAI1* sense: AGGTGATGAGCCTCAACGAGCA, anti-sense: AGTCGTGGTCAGCGTCCAGCT; *ORAI2* sense: CCTGTCGTGGCGGAAGCTCTA, anti-sense: ACTGGTACTGCGTCTCCAGCTG; *ORAI3* sense: TTCCAGCCGCACGTCTGCCTT, anti-sense: CACGGTGGTGCAGGCACTGAA. The relative expression of mRNAs was calculated using the comparative Ct method.

### Flow cytometry

Briefly, gastric cancer cells were transfected as indicated. Forty-eight hours after transfection of RTN2 expressing construct or 72 hours after transfection of *RTN2* siRNA, transfected cells were collected. For examination of cellular apoptosis, after washing with PBS, cells were stained with Propidium Iodide (PI) and fluorescein isothiocyanate (FITC) labelled Annexin V (BD Biosciences), and then they were subjected to flow cytometry for analysis (Beckman Coulter, CA, USA). For detection of cytosolic Ca^2+^, cells were washed and incubated with Fluo-3 AM (3 μM, Beyotime, Nanjing, China) in Ca^2+^-free PBS for 1 hour at 37 °C and protected from light. After rinsing with Ca^2+^-free PBS, the cell suspension was left for 10 minutes at room temperature. Then cells were subjected to flow cytometry.

### Immunofluorescence

Gastric cancer cells were transfected as indicated 48 hours after transfection of RTN2 expressing construct, or 72 hours after transfection of *RTN2* siRNA#1, transfected cells were gathered (10^4^ cells per well) and cultured overnight in 24-well plates with sterile coverslips. For ER Ca^2+^ determination, cells were washed with fresh HHBS and incubated with Mag-Fluo-4 AM working solution (22 μM, AAT Bioquest, China) for 1 hour at 37 °C and protected from light. After rinsing with HHBS, coverslips were mounted to glass slides with antifading solution and sealed with polished nail. For focal adhesion, cells were fixed with 4% paraformaldehyde and stained with primary antibody paxillin-Alexa Fluor 488 (1:1000, Abcam) for 2 hours at 37 °C. After rinsing with PBS, coverslips were mounted to glass slides with antifading solution and sealed with polish nail. Fluorescence images were captured by confocal microscopy.

### Gene set enrichment analysis

We first stratified transcriptome data from The Cancer Genome Atlas Stomach adenocarcinoma (TCGA-STAD) dataset into RTN2 high and low group according to RTN2 median expression and then performed gene set enrichment analysis (GSEA) with Kyoto Encyclopedia of Genes and Genomes (KEGG) pathway sets using the GSEA software.

### Statistical analysis

Analysis was performed with SPSS 22.0 (IBM Corporation, Armonk, NY, USA), GraphPad Prism 5 (GraphPad Software, La Jolla, CA, USA), Stata 12.0 (Stata CorpLP, College Station, TX, USA), and R software version 3.0.2 (R Foundation for Statistical Computing, Vienna, Austria). The relationships between clinical variables and RTN2 expression was analysed by Pearson χ^2^ test. Kaplan–Meier method was used to determine the overall survival and log-rank test was used to compare the overall survival curve between different subgroups. Independent associations between overall survival and assessed clinicopathological predictors were evaluated by multivariate Cox proportional hazards regression models. Differences between the two groups were examined by Student’s two-tailed *t* test. Correlation between two groups was analysed using nonparametric Spearman’s *r* test. Furthermore, R software was utilised to establish a nomogram and the predictive accuracy of this nomogram was tested by calibration plots. All statistical significance was set at two-sided and the *P* value was less than 0.05.

## Supplementary information

Supplementary Information

aj-checklist

Author-contribution-form

Original western blot

## Data Availability

The datasets used and/or analysed during this study are available from the corresponding author on reasonable request.
