# Supplementary material for: Reticulon 2 promotes gastric cancer metastasis via activating endoplasmic reticulum Ca2+ efflux-mediated ERK signalling
Source: Cell Death Dis. 2022 Apr 15;13(4):349. doi: 10.1038/s41419-022-04757-1 (PMC9012842; doi:10.1038/s41419-022-04757-1)
Supplement: Supplementary file 1 — Supplementary Information [file 41419_2022_4757_MOESM1_ESM.docx]

**Supplementary Table 1. Univariate Cox regression analysis of clinicopathological characteristics influencing the overall survival of gastric cancer patients**

|  | **Univariate** | | |
| --- | --- | --- | --- |
| **Factors** | HR | 95% CI | *P*-value |
| **Gender**  female *vs.* male | 1.109 | 0.740-1.662 | 0.616 |
| **Age (years)**  ≥ 60 *vs.* < 60 | 1.515 | 1.043-2.202 | **0.029** |
| **Tumor size (cm)**  > 3.5 *vs.* ≤ 3.5 | 1.891 | 1.301-2.749 | **0.001** |
| **Tumor location**  upper + middle *vs.* lower | 1.103 | 0.742-1.638 | 0.628 |
| **Lauren’s classification**  diffuse + mixture *vs.* intestinal | 1.289 | 0.882-1.883 | 0.190 |
| **Differentiation**  poorly *vs.* well | 1.271 | 0.810-1.996 | 0.298 |
| **Vessel invasion**  present *vs.* absent | 2.580 | 1.639-4.060 | **< 0.001** |
| **Tumor invasion depth**  T3+T4 *vs.* T1+T2 | 2.357 | 1.555-3.571 | **< 0.001** |
| **Lymph node metastasis**  N1+N2+N3 *vs.* N0 | 2.396 | 1.598-3.591 | **< 0.001** |
| **Distant metastasis**  present *vs.* absent | 10.20 | 2.353-44.17 | **0.002** |
| **TNM stage**  III+IV *vs.* I+II | 3.234 | 2.222-4.707 | **< 0.001** |
| **RTN2 expression**  high *vs.* low | 3.401 | 2.330-4.964 | **< 0.001** |

Abbreviations: 95% CI = 95% confidence interval; HR = hazard ratio; TNM = tumor node metastasis. *P*-value < 0.05 marked in bold font shows statistically significant.


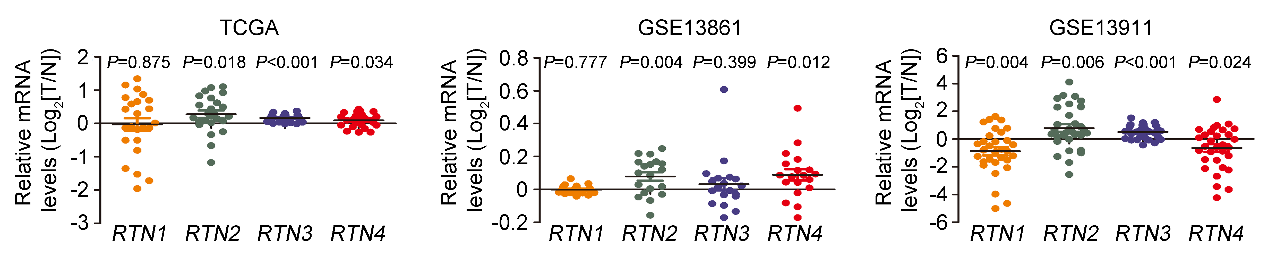


**Supplementary Fig. 1 The mRNA expression patterns of reticulon family in TCGA-STAD, GSE13861 and GSE13911 datasets.** The mRNA expression of *RTN1*-*RTN4* in gastric cancer tumor tissues was compared with that in matched non-tumor tissues.


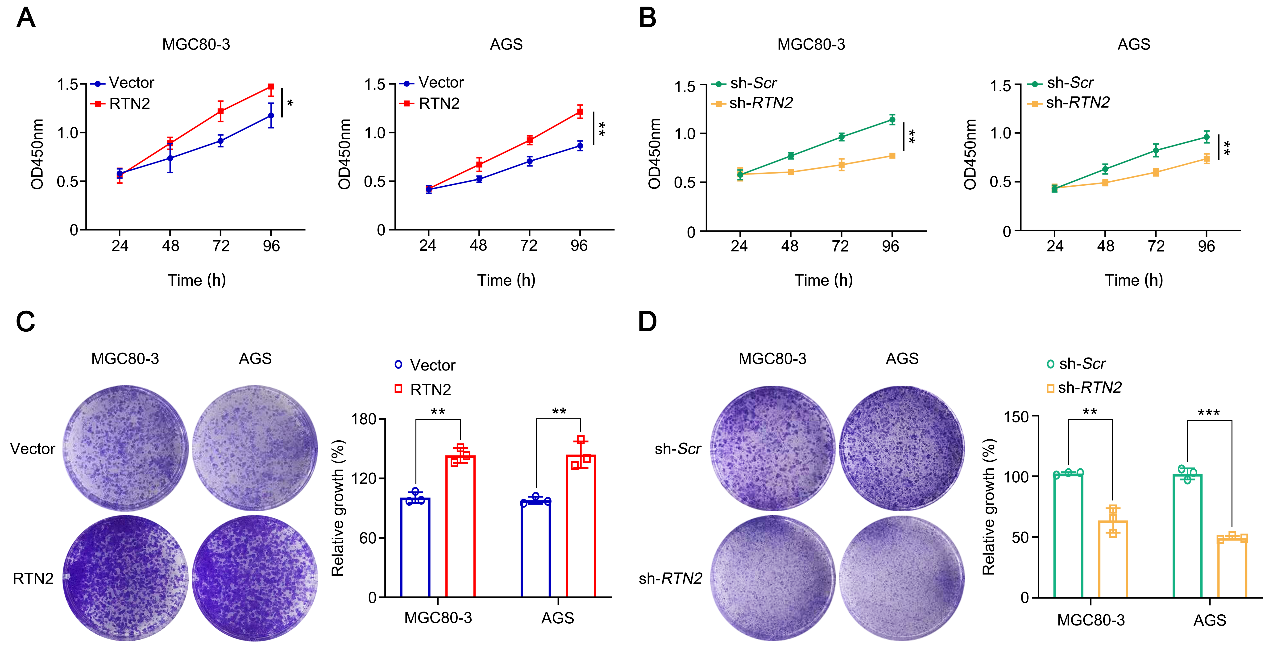


**Supplementary Fig. 2 RTN2 promotes the proliferation of gastric cancer cells. (A-B**) CCK-8 assays were utilized to determine the effects of stable RTN2 overexpression (**A**) or stable knockdown (**B**) on the viability of gastric cancer cells. (**C-D**) Colony formation assays were applied to examine the effects of stable RTN2 overexpression (**C**) or stable knockdown (**D**) on the clonogenic capacity of gastric cancer cells. Results are presented as mean ± SD and statistical significance was calculated by two-way ANOVA analysis (**A-B**) or Student’s two-tailed *t*-test (**C-D**). **, *P* < 0.01; ***, *P* < 0.001.


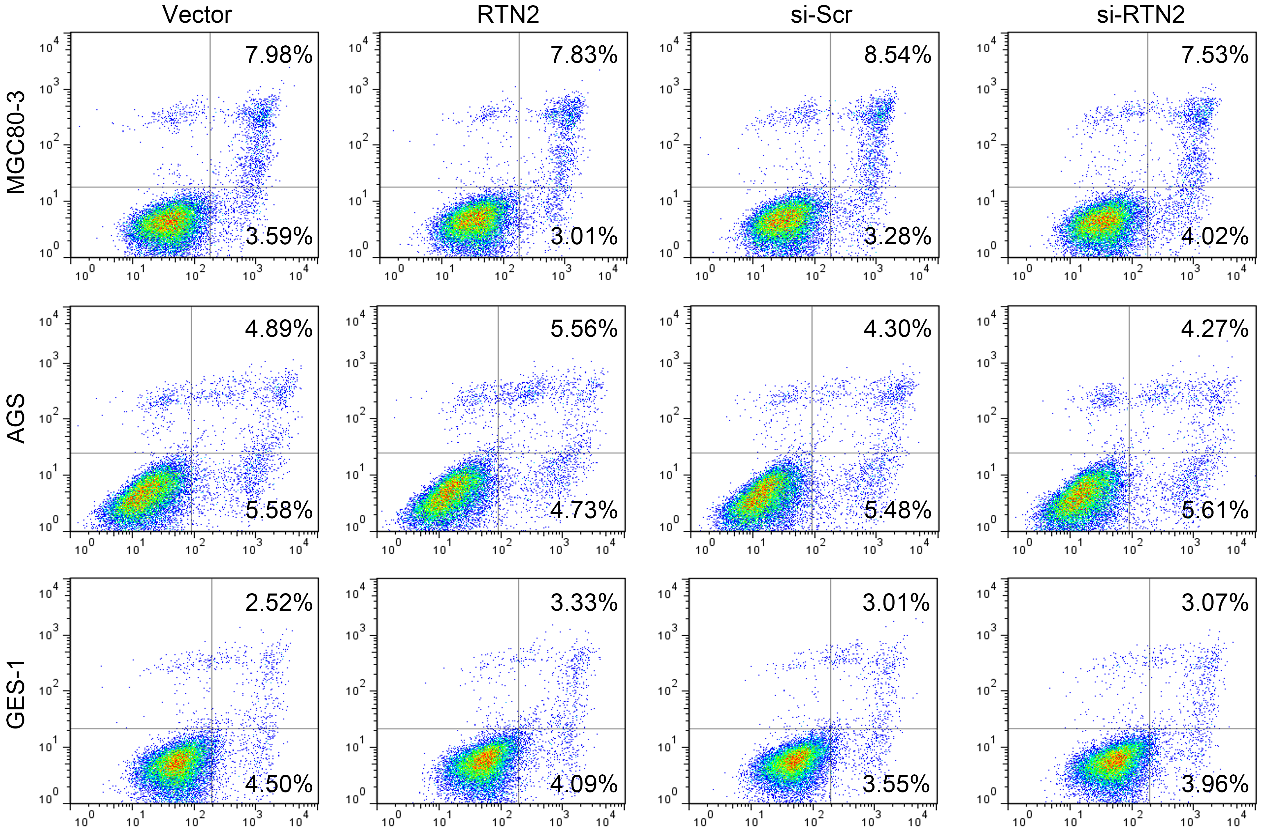


**Supplementary Fig. 3 The influences of RTN2 on cellular apoptosis in gastric cancer cells and normal GES-1 cells.** The apoptosis of MGC80-3, AGS and GES-1 cells was assayed by flow cytometry after staining with Annexin V and PI.


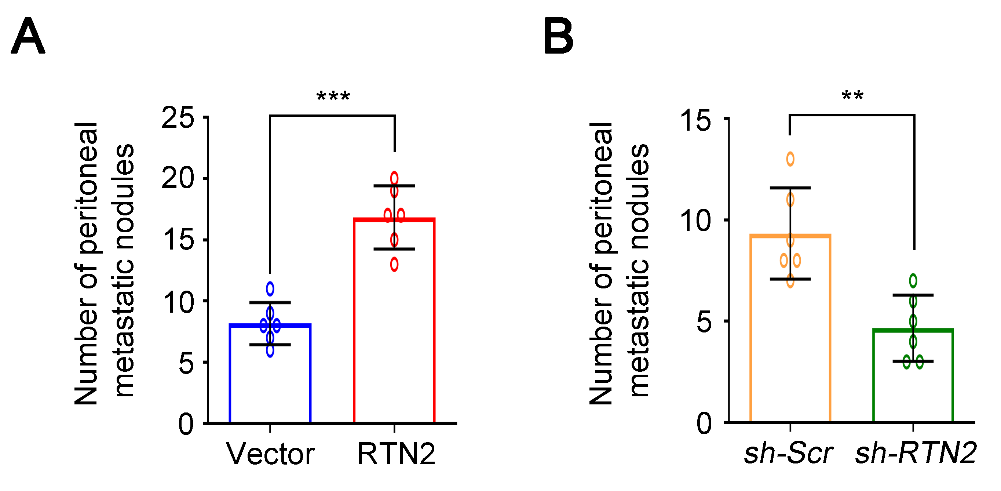


**Supplementary Fig. 4 RTN2 facilitates peritoneal metastasis of gastric cancer cells *in vivo*.** Stable MGC80-3 cells were intraperitoneal injected into mice as described in the “Methods”, and the number of peritoneal metastatic nodules were counted two weeks later. The effects of RTN2 overexpression (**A**) or depletion **(B**) on peritoneal metastasis of gastric cancer cells. Results are presented as mean ± SD, and statistical significance was calculated by Student’s two-tailed *t*-test **(A-B**). **, *P* < 0.01; ***, *P* < 0.001.


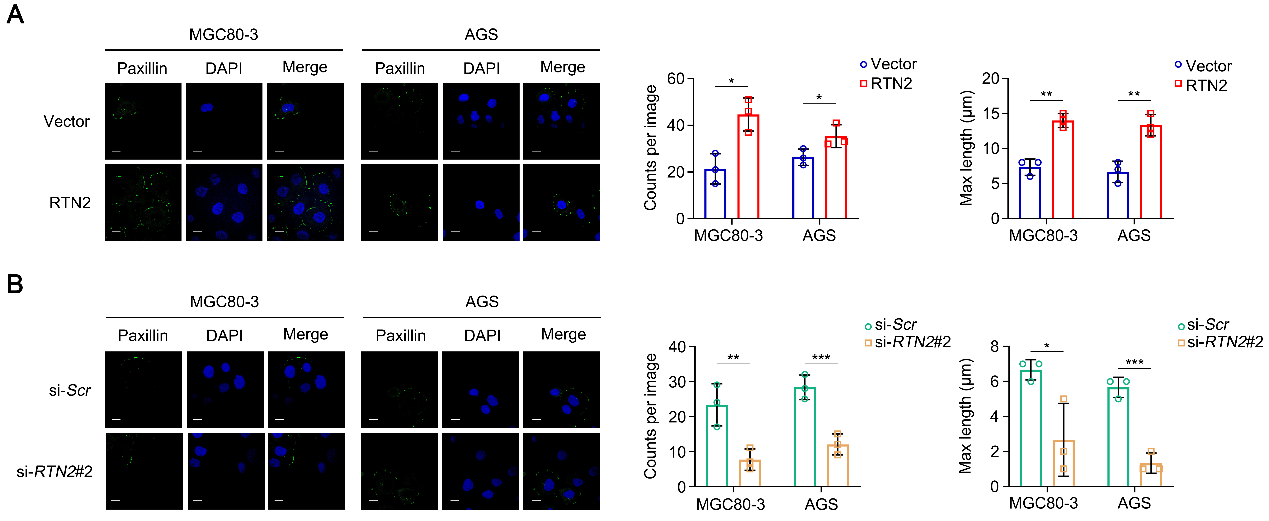


**Supplementary Fig. 5 RTN2 accelerates focal adhesion of gastric cancer cells.** **(A-B**) The effect of RTN2 overexpression (**A**) or depletion (**B**) on paxillin clusters were examined by immunofluorescence staining and the staining count and max length were quantized. Representative images were shown in the left panel. Scale bar, 5 μm. Results are presented as mean ± SD, and statistical significance was calculated by Student’s two-tailed *t*-test **(A-B**). *, *P* < 0.05; **, *P* < 0.01; ***, *P* < 0.001.


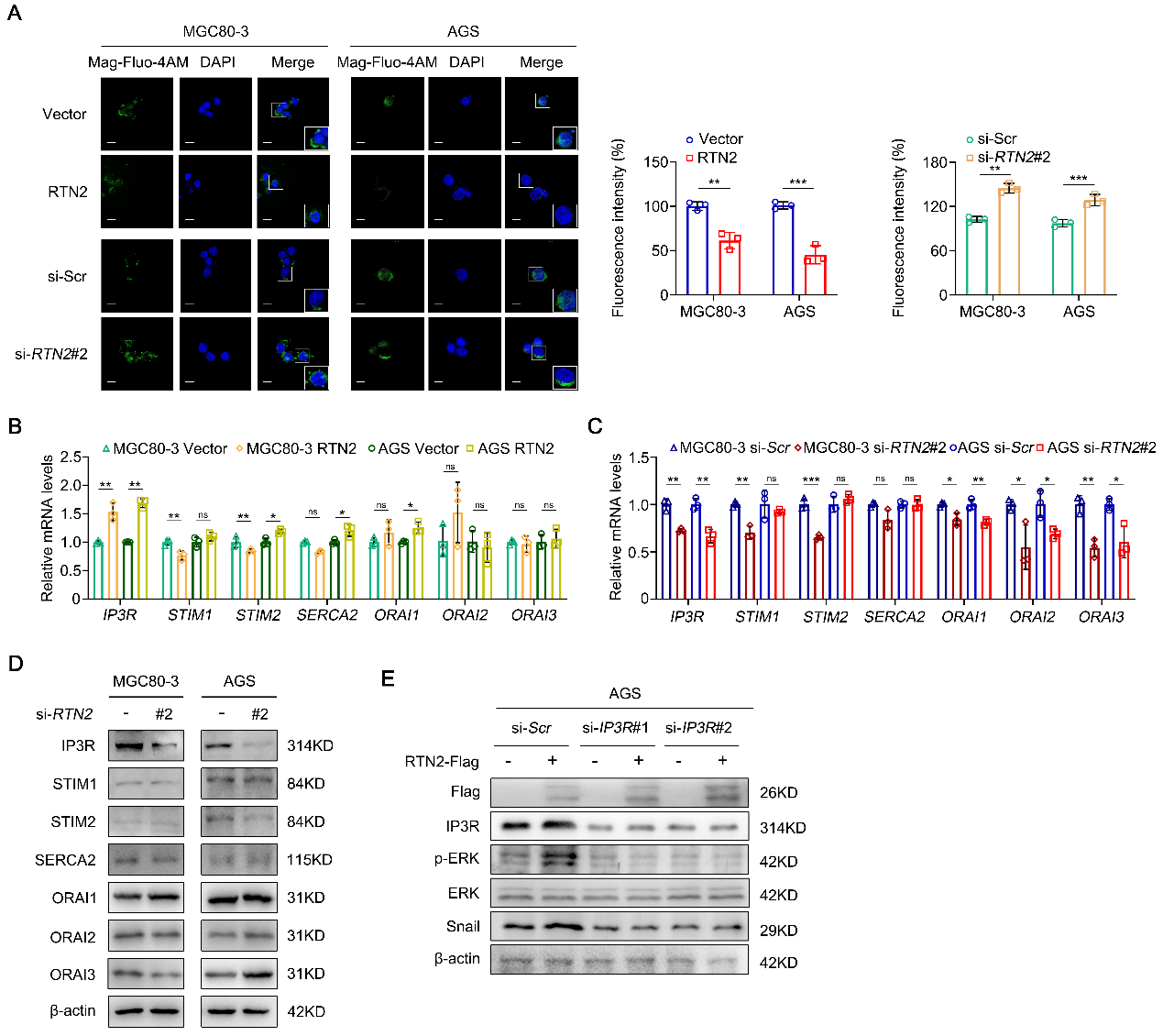


**Supplementary Fig. 6 RTN2 enhances ER** **Ca^2+^ efflux and upregulates IP3R channels in gastric cancer cells.** (**A**) Ca^2+^ content in ER were determined with Mag-Fluo-4 AM staining by confocal microscopy, and the regional enlarged image of Mag-Fluo-4 AM staining was placed on the bottom right corner of representative images. Scale bar, 5 μm. (**B-D**) The effects of RTN2 on Ca^2+^-related channels including IP3R, SERCA2, STIM and ORAI were examined by real-time PCR (**B-C**) and western blot (**D**), respectively. (**E**) Gastric cancer cells were transfected as indicated and applied to western blot. Results are presented as mean ± SD, and statistical significance was calculated by Student’s two-tailed *t*-test (**A-C**). *, *P* < 0.05; **, *P* < 0.01; ***, *P* < 0.001; ns, no significance.


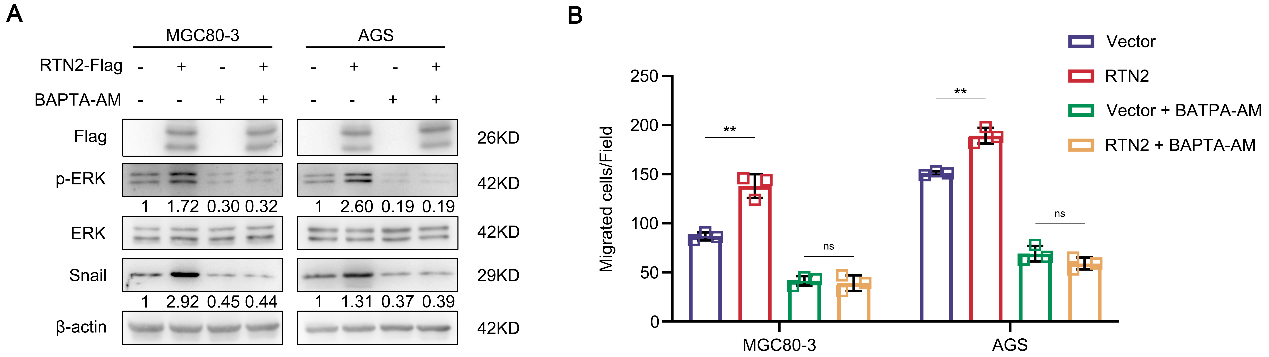


**Supplementary Fig. 7 BAPTA-AM blocks RTN2-induced ERK/Snail signaling activation as well as the migration of gastric cancer cells.** (**A-B**) MGC80-3 and AGS cells were transfected with RTN2 or empty vector, treated with or without BAPTA-AM (10 μM), and then applied to western blot (**A**) and transwell assays (**B**). Results are presented as mean ± SD, and statistical significance was calculated by Student’s two-tailed *t*-test (**B**). **, *P* < 0.01; ns, no significance.
