# Supplementary material for: Reticulon 2 promotes gastric cancer metastasis via activating endoplasmic reticulum Ca2+ efflux-mediated ERK signalling
Source: Cell Death Dis. 2022 Apr 15;13(4):349. doi: 10.1038/s41419-022-04757-1 (PMC9012842; doi:10.1038/s41419-022-04757-1)
Supplement: Supplementary file 3 — Author-contribution-form [file 41419_2022_4757_MOESM3_ESM.pdf]

DECLARATION OF CONTRIBUTIONS TO ARTICLE

ADMC

Manuscript Number: CDDIS-21-1504RRRRR

Journal Name: *Cell Death & Disease* (the 'Journal')

Proposed Title of the Contribution: generate data, design experiments, collect clinical data, statistical analysis, write the manuscript, conceive the project, assemble the figure (the 'Contribution)

Author(s): SS, BL, XZ, YW, HC, HW, JG, XG, YR, HW (the 'Authors')

For all *CDDis* articles, each person named as an author in the published version must be able to show he or she has contributed substantially to the article.

Authorship credit should be based on 1) substantial contributions to conception and design, acquisition of data, or analysis and interpretation of data; 2) drafting the article or revising it critically for important intellectual content; and 3) final approval of the version to be published. Authors should meet conditions 1, 2 and 3.

Any person who cannot be shown to have made a substantial contribution to the article cannot be listed as an author in the final version. The name of any person who is deemed to have made a minor contribution can, however, appear in the Acknowledgments section of the article.

Please complete the table below to indicate the contributions of all named authors to the manuscript.

Author Full Name:	Specification of Contribution to the Manuscript:
Shushu Song	generate data, design experiments, write the manuscript, conceive the project, assemble the figure
Bo Liu	generate data, write the manuscript, assemble the figure
Xiaoqing Zeng	generate data
Hao Chen	collect clinical data
Hao Wu	statistical analysis
Xiaodong Gao	collect clinical data,conceive the project
Jianxin Gu	conceive the project
Yuanyuan Ruan	design experiments, write the manuscript, conceive the project
Hongshan Wang	conceive the project
Yingying Wu	generate data

Please complete the table below to indicate the contributions of all named authors to the figures.

Figure 1:

SS and XZ performed the IHC experiments. HC and XG collected clinical data. HW carried out the statistical analysis. SS assembled the figure.

Figure 2:

HC and XG collected clinical data. HW carried out the statistical analysis. SS assembled the figure.

Figure 3:

SS and YR designed the experiments. SS generated data and assembled the figure.

Figure 4:

SS and YR designed the experiments. SS generated data and assembled the figure.

Figure 5:

SS and YR designed the experiments. SS generated data and assembled the figure.

Figure 6:

SS and YR designed the experiments. BL generate data and assembled the figure.

Signed for and on behalf of the Author(s):	Print Name:	Date:
Yuanyuan Ruan	Yuanyuan Ruan	2021.04.21
