# Supplementary figures and images for: Reticulon 2 promotes gastric cancer metastasis via activating endoplasmic reticulum Ca2+ efflux-mediated ERK signalling
Source: Cell Death Dis. 2022 Apr 15;13(4):349. doi: 10.1038/s41419-022-04757-1 (PMC9012842; doi:10.1038/s41419-022-04757-1)

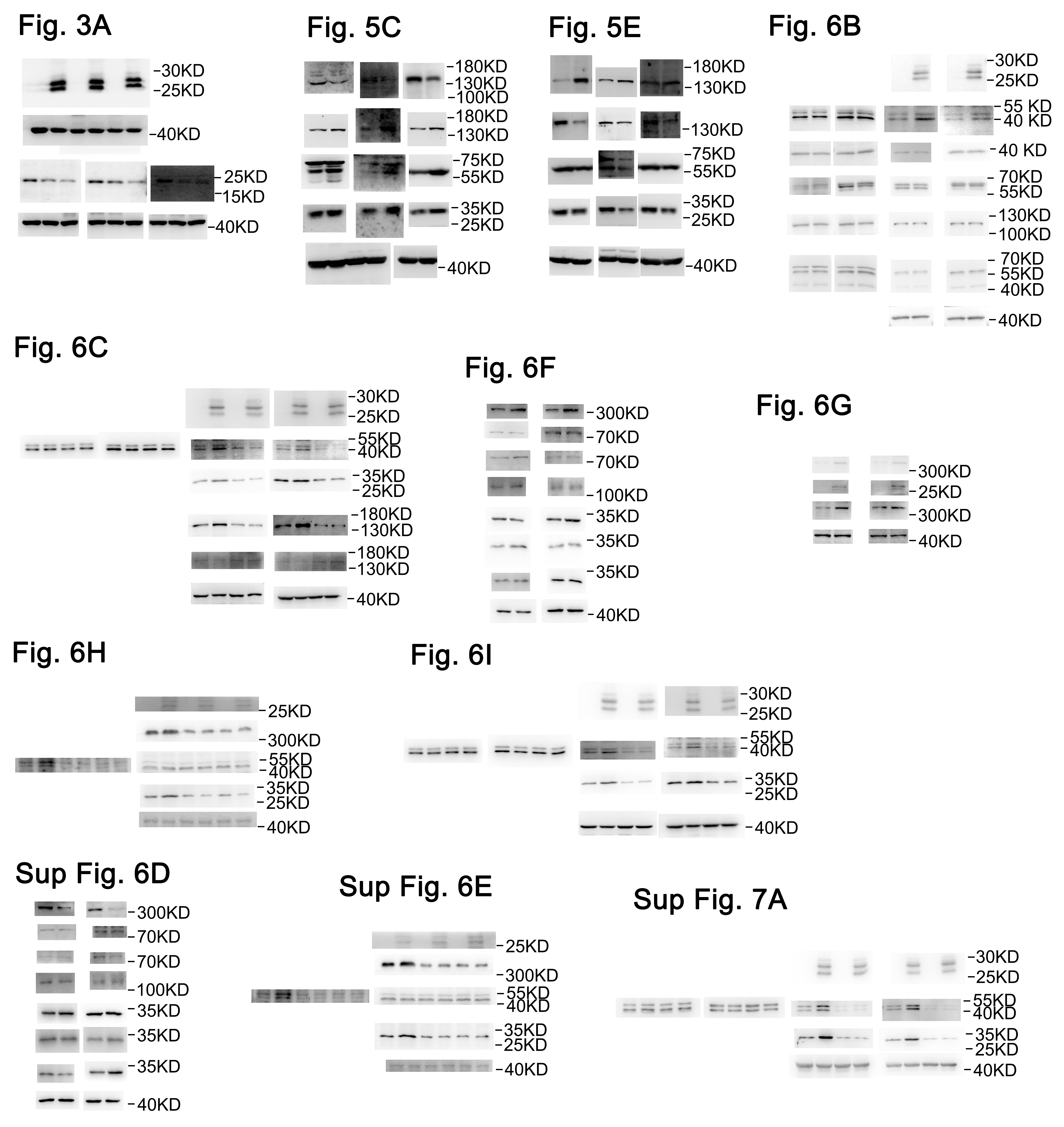

Supplement: Supplementary file 4 — Original western blot [file 41419_2022_4757_MOESM4_ESM.tif]
